# Label-Free In-Situ Estimation of Microparticle Concentration in Micro-Optofluidic Device

**DOI:** 10.3390/s26216736

**Published:** 2026-10-24

**Authors:** Emanuela Cutuli, Lorena Saitta, Giulia Valenti, Gianluca Cicala, Francesca Guarino, Maide Bucolo

**Affiliations:** 1 Department of Industrial, Electrical, Electronic and Computer Engineering, University of Catania, Via Santa Sofia 64, 95125 Catania, Italy; maide.bucolo@unict.it; 2 Department of Civil-Industrial Engineering and Architecture, University of Catania, Via Santa Sofia 64, 95125 Catania, Italy; lorena.saitta@unict.it (L.S.); gianluca.cicala@unict.it (G.C.); 3 Department of Biomedical and Biotechnological Science, University of Catania, Via Santa Sofia 89, 95125 Catania, Italy; giulia.valenti@phd.unict.it (G.V.); francesca.guarino@unict.it (F.G.)

**Keywords:** lab-on-chip, embedded sensing, light scattering, ray tracing, real-time monitoring

## Abstract

In-situ detection and quantification of microparticles remain challenging in embedded lab-on-chip platforms, where limited optical accessibility makes conventional microscope-based analysis impractical. To address this limitation, in this study, a micro-optofluidic platform was developed integrating light delivery and detection directly within the device, thereby enabling label-free concentration estimation without external imaging or sample extraction. The sensing principle exploits concentration-dependent changes in transmitted light caused by microparticle scattering within the microchannel. The platform was evaluated using HL60 cells and PMMA microparticles (CA6) at five concentration levels, ranging from the particle-free condition to $10^{7}\;\mathrm{MPs}\;{\mathrm{mL}}^{-1}$, under coherent laser and LED illumination. Ray-tracing simulations reproduced light propagation and particle–light interactions within the device, predicting particle-dependent differences in the optical flux collected by the detection fiber that were consistent with the experimental response. The measurements revealed a progressive reduction in the transmitted signal with increasing microparticle concentration, with laser illumination providing greater sensitivity and clearer discrimination between the two microparticle types than LED illumination. Three-way ANOVA confirmed that microparticle type, concentration, and illumination source, as well as their interactions, significantly affected the detected optical signal (*p*-value < 0.001). These findings demonstrate that the integrated optical architecture can distinguish concentration-dependent responses across biological and synthetic microparticle suspensions. The proposed platform represents a compact and fully label-free solution for in-situ microparticle monitoring, supporting the future development of self-contained microfluidic sensing platforms.

## 1. Introduction

Lab-on-chip technologies have emerged as versatile platforms for performing complex analytical procedures within miniaturized fluidic environments [1,2,3]. By integrating sample handling, transport, and processing into a single device, these systems enable accurate control of small fluid volumes while reducing sample consumption, reagent requirements, and analysis time [4,5,6,7,8]. These characteristics have promoted their use in a broad range of fields, including biological and clinical analysis [9], pharmaceutical research [10], chemical processing [11], environmental monitoring [12], and industrial quality control [13]. In many of these applications, the microfluidic sample contains suspended entities, such as biological cells or synthetic microparticles, whose characterization provides relevant information on the state, composition, or evolution of the investigated system.

Among the parameters of interest, the concentration of suspended cells or microparticles represents a fundamental indicator in numerous biological and technological applications [14,15]. Cell concentration measurements are required for monitoring proliferation [16], evaluating cell viability and treatment response [17], and monitoring and controlling bioprocesses and microphysiological systems [18]. Similarly, the detection and quantitative characterization of synthetic microparticles are relevant to particle synthesis [19], formulation control [20], filtration, separation or sorting procedures [21].

Analytical strategies for measuring microparticle concentration can be broadly classified as off-chip or on-chip, depending on whether sample characterization is performed outside the device or directly within the microfluidic platform [22,23]. In off-chip analysis, a fluid aliquot is withdrawn from the device and subsequently examined using conventional techniques, such as flow cytometry or spectrophotometry. Although these methods can provide accurate and detailed information, the required sampling introduces a delay between the investigated process and its characterization and generally provides only discrete or endpoint measurements [18]. Sample withdrawal also consumes biological material, may perturb the investigated system, and introduces additional handling steps that can result in variability associated with sample transfer, dilution, contamination, or particle sedimentation [18]. These limitations become particularly relevant in closed microfluidic platforms and long-term cell experiments, where continuous and non-destructive monitoring is desirable [16,18].

Conversely, on-chip approaches integrate the sensing function within the microfluidic platform, enabling measurements to be performed directly in the device while preserving the sample environment [24,25]. This integration can improve temporal resolution, reduce sample handling, and potentially support real-time monitoring and feedback-based control of the investigated process [18,22]. Several physical principles have been exploited for the on-chip detection and characterization of cells and microparticles. Electrical and impedance-based systems measure changes in the electrical properties of the sensing region as individual particles or cells pass between microelectrodes, allowing counting and phenotypic characterization at relatively high throughput [26,27]. However, their response can depend on electrode configuration, particle position, medium conductivity, and electrode–sample interface effects [26,28,29]. Acoustic and acoustofluidic approaches exploit differences in mechanical properties or acoustic contrast to manipulate, separate, or characterize suspended particles, although they commonly require piezoelectric transducers and carefully controlled acoustic fields [30,31]. Electrochemical sensors can provide high sensitivity to specific molecular or cellular targets but typically require electrodes, functionalized interfaces, or recognition elements [18]. Optical methods offer a complementary route because they can interrogate samples rapidly and without physical contact by measuring absorption, fluorescence, transmission, refraction, or scattering [32]. In particular, transmission- and scattering-based measurements can be implemented in a label-free way, without fluorescent or biochemical labels, thereby simplifying sample preparation and reducing the risk that labeling procedures alter the investigated cells or particles [33].

Microscopy remains one of the most widely adopted label-free optical methods for observing, counting, and characterizing microparticles on-chip [34]. Bright-field or phase-contrast microscopy can provide spatially resolved information on particle number, position, morphology, and dynamics, especially when combined with automated image-processing algorithms [35]. Nevertheless, microscopy generally requires a direct and sufficiently transparent optical path between the channel and the external objective, together with a microscope, camera, illumination system, and image-acquisition and processing procedures. These requirements can become restrictive in multilayer or embedded lab-on-chip platforms, in which the microfluidic channel may be located within the device structure, covered by non-transparent components, or positioned outside the working distance of conventional objectives [36]. Moreover, reliance on external imaging instrumentation limits system compactness and portability [37,38,39]. Therefore, an image-free detection strategy that operates locally within the chip is desirable for monitoring microparticle suspensions when direct visualization is impractical or incompatible with the intended device architecture.

Micro-optofluidics provides a suitable framework for addressing these limitations by combining optical and microfluidic components within the same platform. Early investigations demonstrated the potential of optical approaches for real-time blood-flow velocity measurements in microvessels [40] and subsequently explored the integration of polymeric micro-optical interfaces for flow monitoring in biomicrofluidic systems [41]. Optical fibers [42], waveguides [41], lenses [43], and photodetectors [44] can be coupled to, or integrated into, a microfluidic device to deliver and collect light directly across a selected interrogation region. Compared with free-space optical configurations, the relative alignment between the illumination path, the microchannel, and the collection element can be determined by the geometry of the chip itself [45]. This feature can improve measurement reproducibility, reduce the need for repeated manual alignment, and confine the optical measurement to a well-defined volume. In this context, the combined use of ray-tracing simulations and experimental optical characterization has been demonstrated as an effective approach for investigating the influence of optical-component positioning and operating parameters on the response of micro-optofluidic devices, enabling the calibration of fiber-based sensing configurations for in-situ flow monitoring [46]. Moreover, fiber-based integration is particularly attractive because it permits the light source and detector to be located outside the fluidic domain while retaining in-situ optical access to the internal channel [47,48,49].

Several image-free optofluidic approaches have been proposed for detecting and characterizing particles directly within microfluidic devices, with optical fibers providing compact light-delivery and collection paths that reduce the need for free-space optical components [42,47]. Fiber-based flow cytometers and particle counters generally detect transient scattering, absorption, or fluorescence signals generated by individual particles crossing a confined interrogation region [50,51,52,53]. Although these approaches provide single-particle resolution and potentially high throughput, they commonly require hydrodynamic focusing, particle separation, droplet encapsulation, multiple collection paths, or fluorescent labeling to control the particle position and obtain sufficiently discriminative signals [54]. Alternatively, integrated dynamic light scattering and light-extinction spectroscopy have been employed to retrieve particle-size distributions and absolute concentrations from suspensions [55,56]. These techniques enable quantitative particle characterization but generally require multiple collection angles, optical path lengths, correlation analysis, or spectroscopic instrumentation. Integrated lithium-niobate optofluidic platforms have also recently been proposed for the image-free detection of plastic microspheres through their interaction with visible light guided across flowing aqueous droplets [57,58]. These systems demonstrated sensitive microplastic detection using integrated optical waveguides; however, their operation relies on droplet generation and specifically targets synthetic particles dispersed in water. The main characteristics and requirements of these representative approaches are summarized in Table 1.

Despite these advances, comparatively limited attention has been devoted to the label-free and image-free estimation of bulk microparticle concentration in continuous single-phase flow using a simple pair of opposing optical fibers, particularly across both biological cells and synthetic microparticles and under different illumination conditions. The present work addresses this gap by investigating concentration-dependent transmission and scattering without requiring particle focusing, droplet generation, surface functionalization, or sample extraction. Rather than resolving and counting individual particle events, the proposed approach estimates the suspension concentration from the mean optical response measured over a defined in-situ interrogation volume.

In this work, a micro-optofluidic platform is proposed for the label-free and in-situ estimation of microparticle concentration in a microchannel without microscope-based image acquisition or sample extraction. The device incorporates opposing optical fibers for light delivery and collection, defining an optical interrogation volume directly within the flowing suspension. Its response is investigated using HL60 cells and CA6 synthetic microparticles over five concentration levels under coherent laser and LED illumination. A ray-tracing model is employed to analyze light propagation and particle-induced redistribution within the interrogation region, whereas experimental measurements are used to characterize the concentration-dependent transmitted signal. Finally, a three-way analysis of variance (ANOVA) is performed to quantify the effects of microparticle type, concentration, illumination source, and their interactions. By combining ray-tracing simulations, systematic experimental characterization, and multifactor statistical analysis, this study demonstrates and quantitatively characterizes the capability of the proposed architecture for label-free, image-free, and in-situ microparticle concentration estimation, supporting its future implementation in embedded microfluidic platforms.

The paper is organized as follows. Section 2 describes the design, working principle, and fabrication of the micro-optofluidic device (Section 2.1), together with the sample preparation procedure (Section 2.2), the experimental setup (Section 2.3) and campaign (Section 2.4), and the ray-tracing model (Section 2.5). Section 3 presents and discusses the numerical and experimental results, including the simulated optical response (Section 3.1), the concentration-dependent measurements obtained under laser and LED illumination (Section 3.2), and the three-way ANOVA (Section 3.3). Finally, Section 4 summarizes the main conclusions and outlines potential directions for future development.

## 2. Materials and Methods

### 2.1. Micro-Optofluidic Device: Design, Working Principle and Fabrication

**Figure 1:**
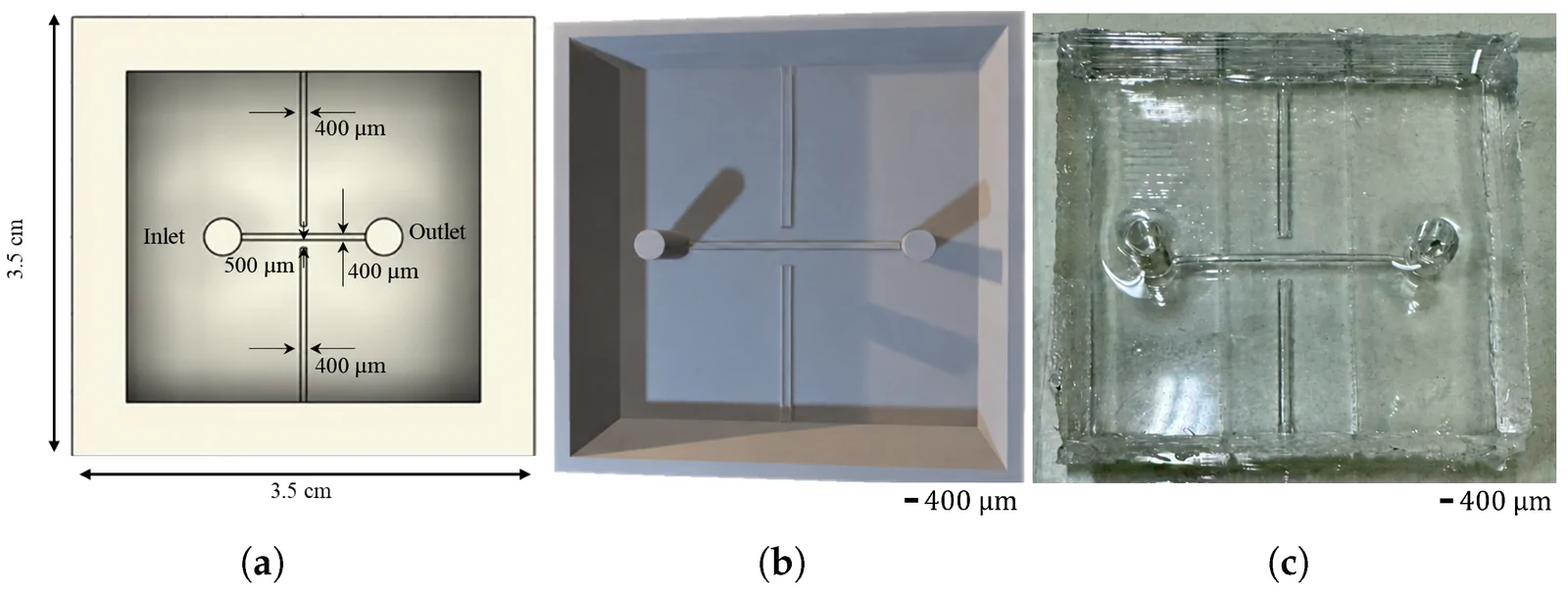
Micro-optofluidic device design and fabrication: (**a**) schematic geometry of the microfluidic and optical channels, (**b**) corresponding 3D printed mold, and (**c**) resulting PDMS device.

The micro-optofluidic (MoF) device investigated in this study has global planar dimensions of $\left(3.5\times 3.5\right)\;{\mathrm{cm}}^{2}$ and features a straight microfluidic channel with a width of 400 μm, connected to one inlet and one outlet for sample injection and discharge, respectively. Two 400 μm-wide transverse slots are designed to accommodate the optical fibers and end 500 μm from the microchannel walls without intersecting the fluidic channel, as illustrated in Figure 1a.

**Figure 2:**
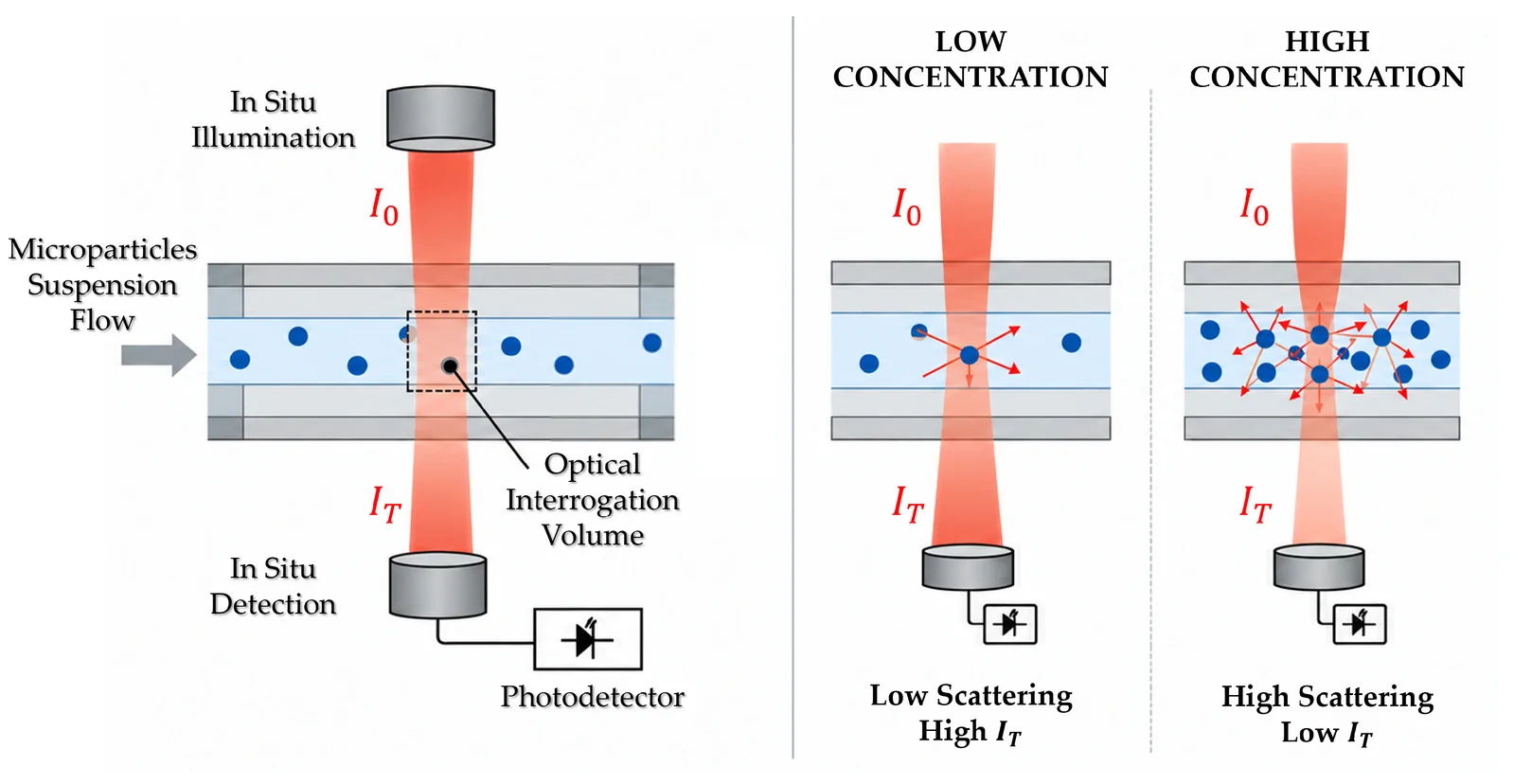
Working principle schematization of the proposed micro-optofluidic device at low and high concentrations of dispersed phase (microparticles and/or cells).

The working principle of the proposed device is schematically illustrated in Figure 2. The microparticle suspension flows through the microchannel and crosses the optical interrogation volume defined by the in-situ illumination and detection fibers. The illumination fiber, coupled to a light source, delivers light with an incident intensity $I_{0}$ to the optical interrogation volume. Upon interaction with the suspended microparticles, part of the incident light is scattered, while the transmitted component, with intensity $I_{T}$, is collected by the opposing detection fiber coupled to a photodetector. Under fixed illumination and geometrical conditions, an increase in microparticle concentration produces a greater number of light–particle interactions, thereby enhancing scattering and reducing $I_{T}$. At low concentrations, the limited scattering results in a higher transmitted intensity, whereas at high concentrations, the increased scattering produces a lower detected signal. Therefore, the measured transmitted intensity provides a concentration-dependent optical response. It should be noted that, although scattering and reflection contribute to the redistribution of incident light, their individual contributions were not separately quantified in the present study. Indeed, both the experimental measurements and the ray-tracing simulations focused on the optical intensity collected along the detection path, rather than on the angular distribution of scattered and reflected radiation. A quantitative discrimination of these contributions would require dedicated angularly resolved optical measurements, potentially complemented by integrating-sphere-based characterization [59,60,61]. From a numerical perspective, a more comprehensive analysis would require detailed wavelength-dependent optical properties of the dispersed phase and surrounding medium, appropriate scattering models accounting for particle morphology and refractive index heterogeneity, and dedicated ray-path and flux analyses [60,62]. Therefore, the present results establish a concentration-dependent variation in the collected optical response without quantitatively attributing signal attenuation to individual light-interaction mechanisms.

**Figure 3:**
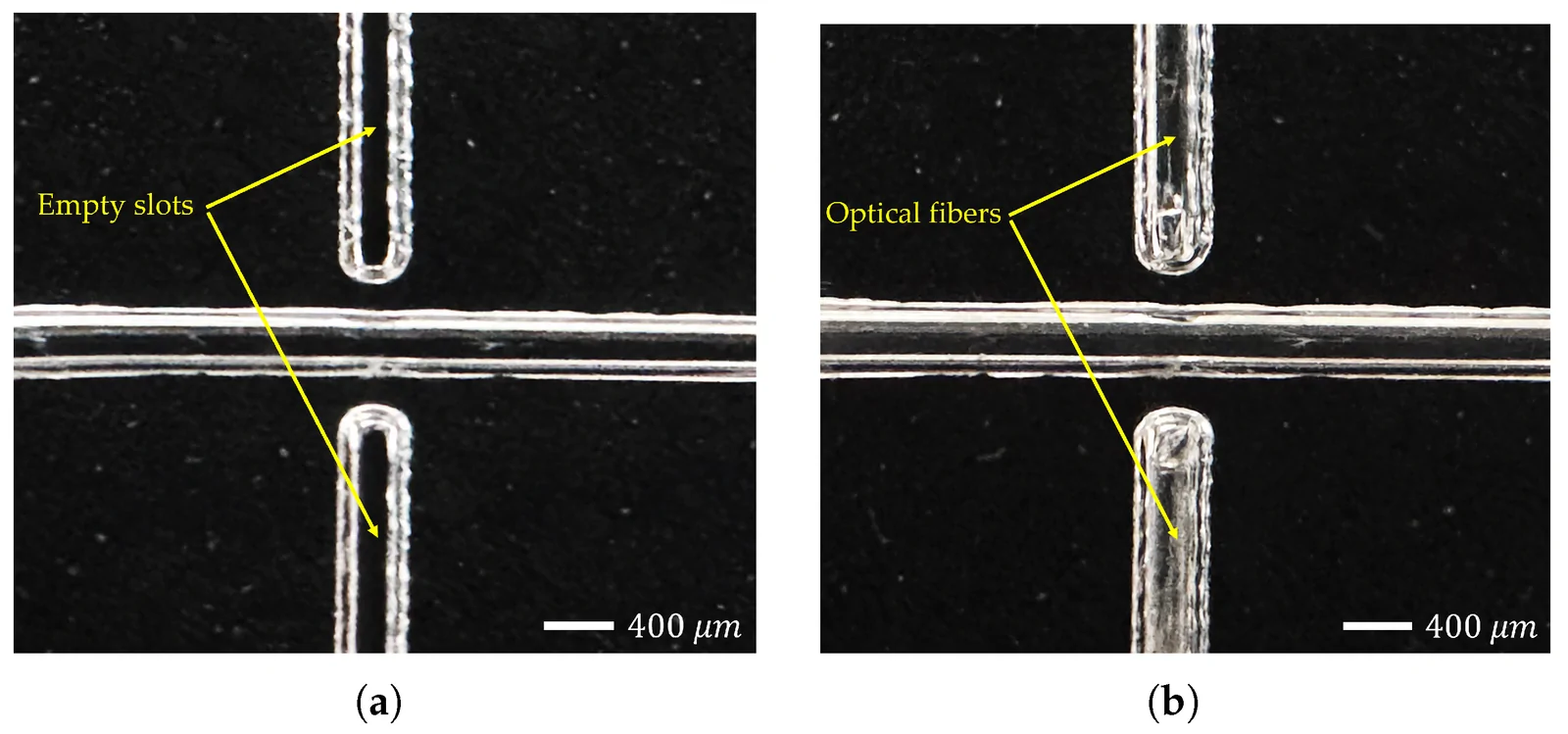
Actual images of the PDMS device showing (**a**) the empty optical slots and (**b**) the optical fibers inserted into the slots.

The MoF devices were fabricated using polydimethylsiloxane (PDMS) through a master–slave approach based on Inkjet 3D printing, elsewhere described in detail [63,64,65]. The mold for the PDMS casting was fabricated using a Stratasys Objet260 Connex1 (Stratasys, Los Angeles, CA, USA) 3D printer and VeroWhitePlus RGD835 photopolymer resin (OVERMACH S.p.A., Parma, Italy), a proprietary blend of acrylate monomers and photoactivators. The 3D printed mold is shown in Figure 1b. The PDMS used for device fabrication was the SYLGARD™184 Silicone Elastomer kit (Dow Corning, Midland, MI, USA), consisting of a base and curing agent, purchased from Farnell Italia S.R.L. (Milan, Italy). PDMS was prepared by mixing the silicone elastomer base and curing agent with a mixing ratio of 10:1 for the device layer and 5:1 for the cover layer, then degassed, poured into the mold, and cured at room temperature for 48 h. The cured parts were demolded and bonded, with both surfaces plasma-treated for 3 min using Elveflow’s Plasma Bonding Pen (Elveflow, Paris, France) to enhance adhesion. The final bonded device is shown in Figure 1c, while Figure 3 provides detailed views of the optical slots before (see Figure 3a) and after (see Figure 3b) fiber insertion, respectively, illustrating the positioning of the two opposing optical fibers on either side of the microchannel.

### 2.2. Sample Preparation

Two types of microparticles were investigated in this study: HL60 cells, with an average diameter of 15 μm and Spheromers^®^ CA6 synthetic microparticles (Lamberti S.p.A., Gallarate, Italy), with a nominal diameter of 6 μm. Their main physical properties are summarized in Table 2.

HL60 cells were cultured in RPMI-1640 medium supplemented with 20% fetal bovine serum (FBS) and maintained at 37 °C in a humidified incubator with 5% CO_2_. Before the experiments, the cells were collected and resuspended in phosphate-buffered saline (PBS).

CA6 microparticles were suspended in a glycerol–deionized water mixture at an 80:20 volumetric ratio. This carrier fluid was selected to approximate the density of the microparticles, thereby limiting sedimentation and promoting a homogeneous distribution throughout the experiments, similar to previous studies [63]. Before each dilution step, the CA6 suspensions were gently vortexed to ensure uniform microparticle dispersion, whereas the HL60 suspensions were gently mixed to preserve cell integrity.

For each microparticle type, four non-zero concentration levels were prepared: $10^{4}$, $10^{5}$, $10^{6}$, and $10^{7}$ microparticles per mL. A particle-free solution (0 microparticles per mL) was also tested as a reference, resulting in five experimental concentration conditions for each class. Each suspension was prepared with a final volume of 5mL distribution.

### 2.3. Experimental Setup

The experimental setup employed in this study comprises: (i) a hydrodynamic actuation system for introducing the microparticle suspension into the micro-optofluidic device; (ii) an illumination system coupled to the input optical fiber; (iii) the MoF device positioned under an optical microscope; (iv) an optical detection system consisting of an output optical fiber coupled to a photodiode; and (v) computer-based systems for optical monitoring and signal acquisition.

To facilitate the positioning of the optical fibers, the MoF device was placed on the stage of an OPTIKA IM-300 Phi optical microscope (OPTIKA, Ponteranica, BG, Italy) equipped with a 10× objective lens. The microscope was used to monitor and verify the insertion and alignment of the illumination and detection fibers within the corresponding slots. It was equipped with a 340M Fast Frame CCD camera (Thorlabs, Newton, NJ, USA), providing a resolution of 640×480 pixels with a pixel size of 7.4 μm. The camera was connected to a computer via USB and operated using ThorCam™ software (version 3.7.0).

The microparticle suspensions were injected into the microchannel using a Fusion 4000 syringe pump (Chemyx, Stafford, TX, USA).

Two illumination conditions were investigated. Coherent illumination was provided by a NovaPro 660-125 laser system (RGB Lasersystems, Kelheim, Germany), operating at a wavelength of 660nm and an optical power of 1mW. Broadband illumination was instead provided by a fiber-coupled white LED source (Thorlabs, Newton, NJ, USA). The selected source was coupled to an input optical fiber with a core diameter of 365 μm, which delivered the light to the optical interrogation region of the device.

**Figure 4:**
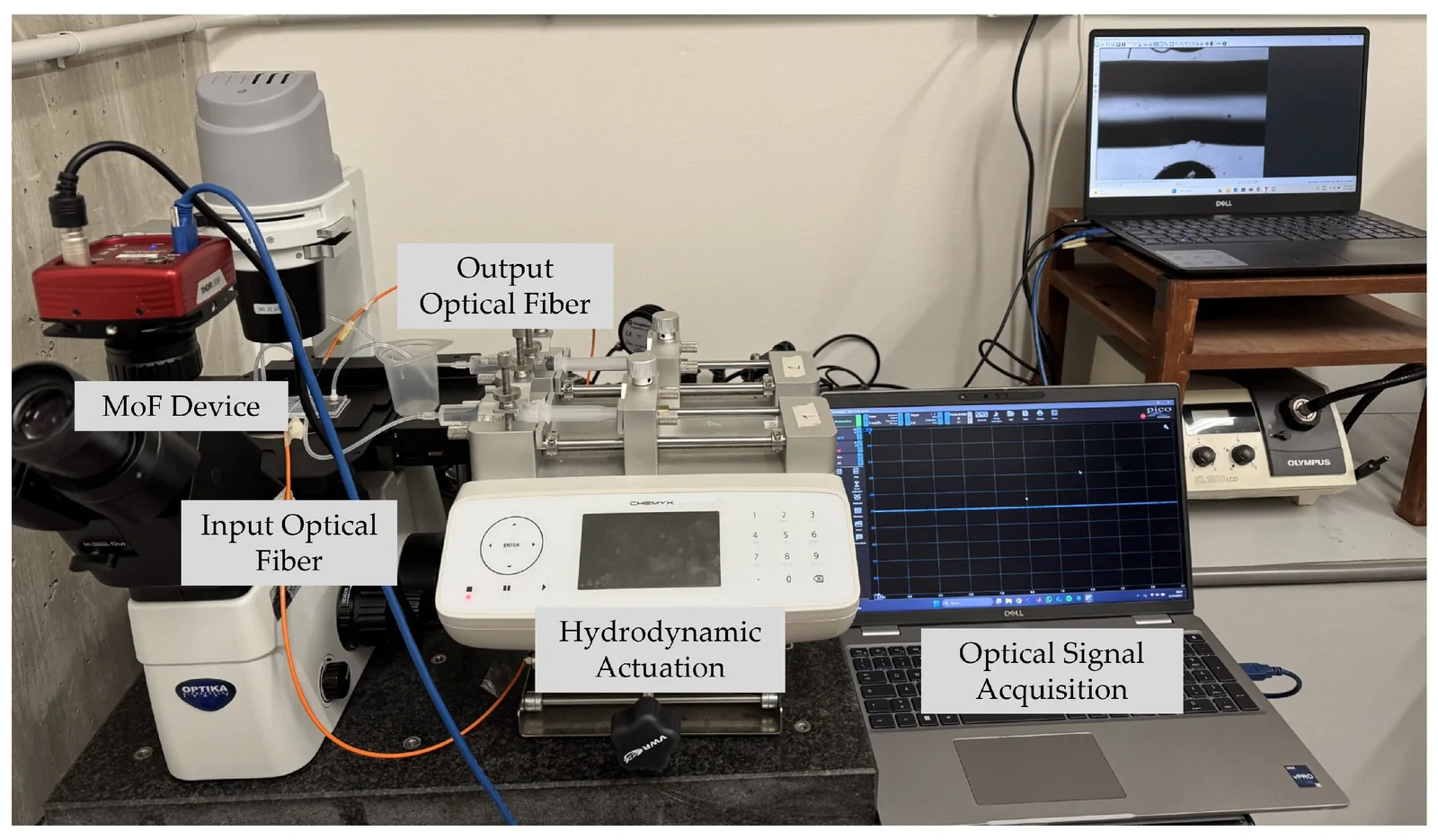
Real picture of the experimental setup, including the micro-optofluidic device, input and output optical fibers, hydrodynamic actuation system, and optical signal acquisition system.

The transmitted light was collected by an output optical fiber with a core diameter of 365 μm and detected using a PDA100A photodiode (Thorlabs, Newton, NJ, USA) with the gain set to 40dB. The photodiode output was recorded using a PicoScope 2204A USB oscilloscope (Pico Technology, Cambridgeshire, UK) operating at a sampling rate of 1.5 kHz. A photograph of the real experimental setup is reported in Figure 4.

### 2.4. Experimental Campaign and Data Analysis

The experimental campaign was conducted using the two microparticle types, CA6 synthetic microparticles and HL60 cells, at five concentration levels: 0, $10^{4}$, $10^{5}$, $10^{6}$, and $10^{7}$ particles or cells per mL. The zero-concentration condition corresponded to the particle-free carrier fluid used for each microparticle type. Before introducing the microparticles, the optical system was calibrated to obtain comparable background signals for PBS and the glycerol–water solution. This calibration minimized the contribution of the different carrier media to the detected signal, allowing the subsequent variations to be primarily associated with the presence and concentration of the microparticles.

All suspensions were injected into the microchannel at a constant flow rate of $0.008\;\mathrm{mL}\;\min^{-1}$. This flow rate was selected as a compromise between limiting particle sedimentation and ensuring a sufficient residence time within the optical interrogation volume. Considering the (400×400) μm^2^ channel cross-section, the imposed flow rate corresponds to a nominal mean fluid velocity of approximately $0.83\;\mathrm{mm}\;s^{-1}$. Accordingly, the estimated mean residence time within the 400 μm-long optical interrogation region is approximately 0.48s.

Each microparticle type–concentration combination was tested under both coherent laser illumination and broadband white LED illumination. For each experimental condition, R = 3 replicated acquisitions of 60s were performed at a sampling frequency of 1.5kHz, resulting in 90,000 samples per acquisition. The experiments were conducted under ambient laboratory conditions. The laboratory temperature was monitored throughout the experiments and maintained between 20 °C and 25 °C. The experimental campaign is reported in Table 3.

Optical signal post-processing was performed in MATLAB R2023b (MathWorks, Natick, MA, USA). The raw signal acquired by the digital oscilloscope was first low-pass filtered using a cutoff frequency of 40Hz to attenuate high-frequency components. A subsequent smoothing procedure was applied to further reduce signal fluctuations. For each acquisition, the mean optical signal and its standard deviation were then calculated as

(1) $${\bar{S}}_{\mathrm{opt}}=\frac{1}{N}\sum_{n=1}^{N}S_{\mathrm{opt}}\left(n\right),$$

where *N* = 90,000 is the number of samples collected during each acquisition. The mean value ${\bar{S}}_{\mathrm{opt}}$ was used to evaluate the concentration-dependent optical response.

The three values of ${\bar{S}}_{\mathrm{opt}}$ obtained from the repeated acquisitions under each experimental condition were subsequently averaged as

(2) $$\langle {\bar{S}}_{\mathrm{opt}}\rangle =\frac{1}{R}\sum_{r=1}^{R}{\bar{S}}_{\mathrm{opt},r},$$

and the corresponding standard error of the mean (SEM) was calculated as

(3) $$\mathrm{SEM}=\frac{s_{{\bar{S}}_{\mathrm{opt}}}}{\sqrt{R}},$$

where R=3 is the number of repeated acquisitions and $s_{{\bar{S}}_{\mathrm{opt}}}$ is the sample standard deviation of the three acquisition means. The resulting $\langle {\bar{S}}_{\mathrm{opt}}\rangle$ and SEM were used to report the optical response for each combination of microparticle type, concentration, and illumination source.

Prior to the experimental campaign, a preliminary thermal assessment, by using a SLF3S-1300F sensor (Sensirion, Stäfa, Switzerland), was performed to verify that laser illumination did not induce appreciable heating effects that could potentially compromise temperature-sensitive biological samples. To this aim, deionized water was circulated through the microfluidic channel, and its temperature was monitored over 60 s of continuous laser illumination at input powers of 1, 5, and 10 mW. The recorded temperatures were 23.0±0.2 °C at 1 mW, 22.9±0.2 °C at 5 mW, and 22.9±0.2 °C at 10 mW (mean ± standard error). The substantial stability of these values indicated no appreciable laser-induced heating under the investigated conditions, supporting the subsequent experimental campaign and the potential applicability of the proposed optical monitoring approach to biological samples.

### 2.5. Ray-Tracing Model

**Figure 5:**
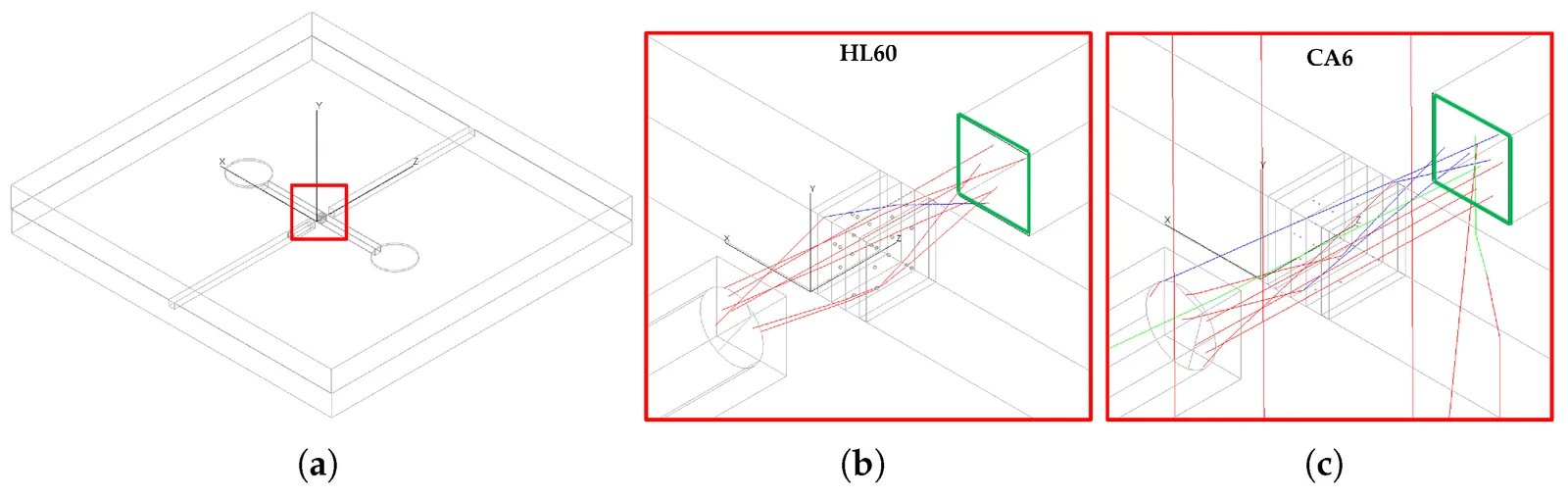
TracePro ray-tracing model of the micro-optofluidic device: (**a**) complete device geometry, with the optical interrogation region highlighted in red and ray propagation through the interrogation volume containing (**b**) HL60 cells and (**c**) CA6 microparticles. The entrance face of the detection fiber, outlined in green, was defined as the receiver surface. Ray colors indicate the optical flux carried by each ray relative to its initial flux at emission: red (*high*, 66.7–100%), green (*intermediate*, 33.3–66.7%), and blue (*low*, 0–33.3%) ([66], **Section: Flux-based ray colors**).

The optical simulations were performed using TracePro (Lambda Research Corporation, Littleton, MA, USA) [66], based on a non-sequential Monte Carlo ray-tracing approach. The simulation environment reproduced the complete geometry of the micro-optofluidic device, including the microchannel, optical slots, and illumination and detection fibers, as shown in the left panel of Figure 5a. The corresponding geometrical, material, and optical properties were assigned to each domain. The portion highlighted by the red box identifies the optical interrogation region considered in the simulations.

The optical interrogation volume was modeled as a (400×400×400) μm^3^ region within the microchannel. A single microparticle concentration of $3\times 10^{5}\;\mathrm{MPs}\;{\mathrm{mL}}^{-1}$ was selected to isolate the effect of microparticle type on light propagation. This value lies approximately in the central region, on a logarithmic scale, of the non-zero concentration range investigated experimentally, i.e., $10^{4}$–$10^{7}\;\mathrm{MPs}\;{\mathrm{mL}}^{-1}$ (see Table 3). Since the modeled volume corresponds to $6.4\times 10^{-5}\;\mathrm{mL}$, the selected concentration resulted in ∼20 microparticles within the optical interrogation volume. Separate simulation assemblies were generated for HL60 cells and CA6 microparticles while maintaining the same concentration and number of microparticles, as shown in Figure 5b,c. To isolate the effect of microparticle type, identical spatial coordinates were used in the HL60 and CA6 simulation assemblies. Their diameter, density, surface roughness, and refractive index were assigned according to the physical properties reported in Table 2.

**Figure 6:**
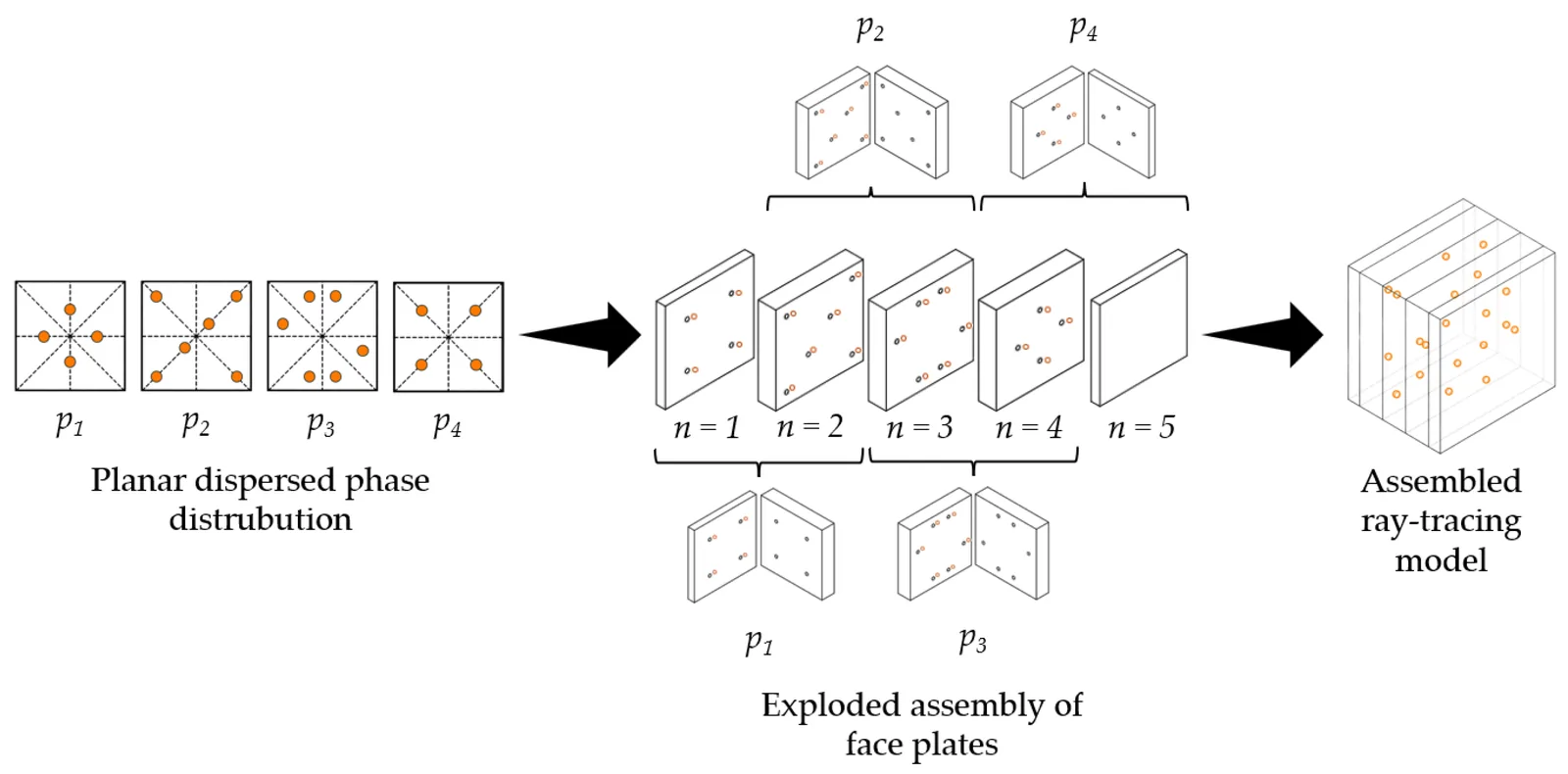
Geometrical modeling and assembly procedure for the ray-tracing simulation optical interrogation volume containing 20 HL60 cells or CA6 MPs (named dispersed phase): definition of four planar particle distribution patterns $p_{1},\ldots ,p_{4}$ (left panel); construction and assembly of five face plates (n=1,…,5) incorporating hemispherical cavities and corresponding spherical elements (central panel); and resulting assembled three-dimensional optical interrogation model (right panel).

The optical interrogation model was modeled as follows. The three-dimensional model used for the ray-tracing simulations was developed in Autodesk^®^ Fusion 360, considering an optical interrogation volume containing 20 spherical elements representing the dispersed phase, that is the HL60 cells or CA6 MPs. As illustrated in the central panel of Figure 6, five face plates (n=1,…,5) were designed and assembled to define four intermediate planes ($p_{1},\ldots ,p_{4}$), each containing a certain number of spherical particles. The particle positions were deterministically defined through four specifically designed planar distribution patterns, rather than randomly generated, to prevent particle overlap and to maximize the spatial coverage of the optical interrogation cross-section (see left panel of Figure 6). Each face plate incorporated hemispherical cavities at the prescribed particle locations, with diameters of 15 μm for HL60 cells and 6 μm for CA6 MPs. Corresponding solid spheres were then positioned within these cavities to represent the dispersed phase. Finally, the five face plates and spherical elements were assembled into the complete three-dimensional model used for optical ray-tracing simulations (see right panel of Figure 6). Accordingly, the reported simulated flux values refer to this predefined spatial distribution of 20 particles, without averaging over multiple randomly generated particle configurations.

The optical fibers were modeled according to the nominal opto-geometrical specifications provided by the manufacturer, including a core diameter of 365 μm and a numerical aperture of NA=0.22. To reproduce the experimental laser condition, the optical source was modeled as a monochromatic flux source with a wavelength of 660nm and an optical power of 1mW. A Lambertian angular distribution confined within the divergence cone determined by the fiber numerical aperture was assigned to the source, which was discretized into 100 rays. The rays were propagated through the illumination fiber, the PDMS structure, and the particle-containing interrogation volume toward the opposing detection fiber. The entrance face of the detection fiber, outlined in green in Figure 5b,c, was defined as the receiver surface. Irradiance maps were generated over this surface, and the total incident optical flux was calculated to quantify the light collected by the detection fiber. The irradiance distributions and collected flux values obtained for the HL60 and CA6 models were subsequently compared to assess the influence of microparticle type and its physical properties on the transmitted optical response.

## 3. Results and Discussion

### 3.1. Ray-Tracing Simulation

**Figure 7:**
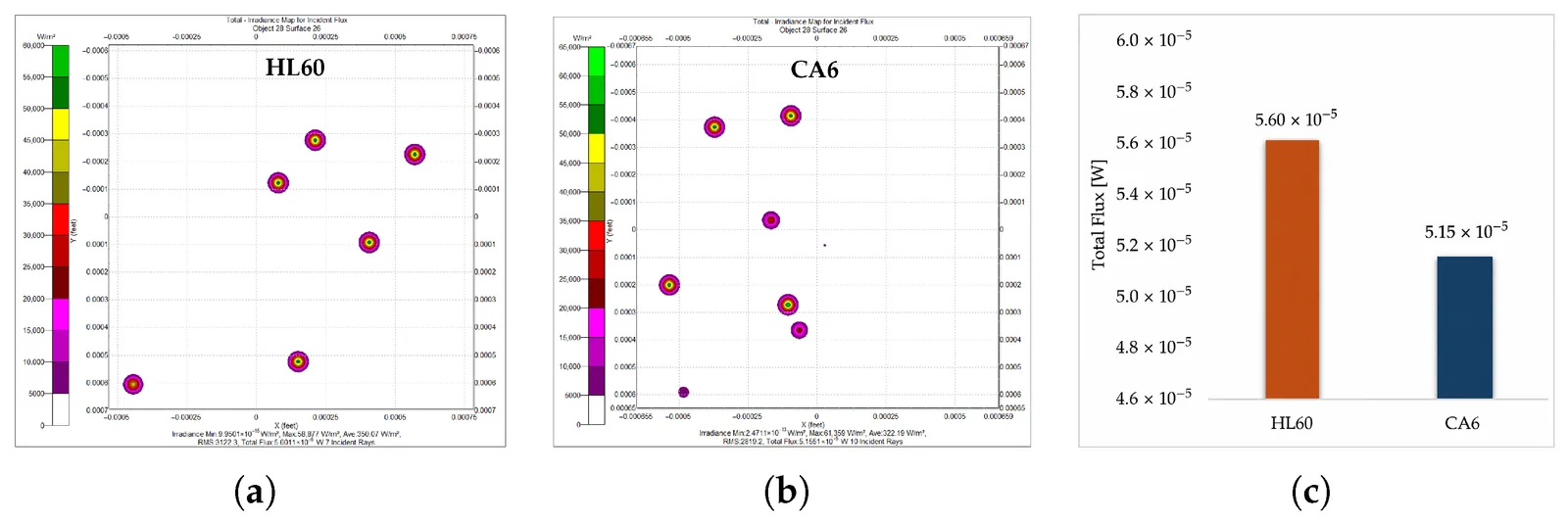
Simulated irradiance maps on the entrance surface of the detection fiber for (**a**) HL60 cells, (**b**) CA6 microparticles, and (**c**) bar plot of the corresponding total incident optical flux collected by the detection fiber.

The ray-tracing results provide a preliminary interpretation of the interaction between the incident light and the two microparticle suspensions before considering the experimental measurements. The different ray trajectories obtained for the HL60 and CA6 models, shown in Figure 5b,c, indicate that the spatial redistribution of the optical power depends on the properties assigned to the microparticles and their surrounding medium. In particular, the CA6 configuration exhibits a more pronounced angular redistribution of the rays, with a larger fraction of the optical paths deviating from the direct source–receiver direction. The corresponding irradiance distributions on the entrance face of the detection fiber are reported in Figure 7a,b for HL60 cells and CA6 microparticles, respectively. Both maps are characterized by localized irradiance peaks associated with the individual ray intersections with the receiver surface. As shown in Figure 7c, the total flux reaching the receiver surface was $5.60\times 10^{-5}\;W$ for HL60 cells and $5.15\times 10^{-5}\;W$ for CA6 microparticles. Therefore, at the same concentration, the CA6 suspension produced an approximately 8.0% reduction in collected optical power relative to the HL60 suspension. This difference cannot be attributed exclusively to particle diameter. Although the nominal diameter of the HL60 cells is larger than that of the CA6 microparticles, the simulations predict a higher transmitted flux for the HL60 model. The optical response is instead determined by the combined effects of particle size, the refractive-index contrast between each microparticle and its suspending medium, and the assigned surface properties. For the properties reported in Table 2, the combined optical contrast and surface interaction of the CA6 system result in a greater loss of power from the collection area, overcoming the effect expected from its smaller particle diameter. Particle density, conversely, does not directly govern ray propagation in the static optical model; its relevance is primarily associated with sedimentation and particle distribution under experimental conditions.

These results support the sensitivity of the proposed micro-optofluidic configuration to particle-dependent scattering and provide a physical basis for interpreting the concentration-dependent experimental measurements presented in the following sections.

### 3.2. Concentration-Dependent Optical Response

**Figure 8:**
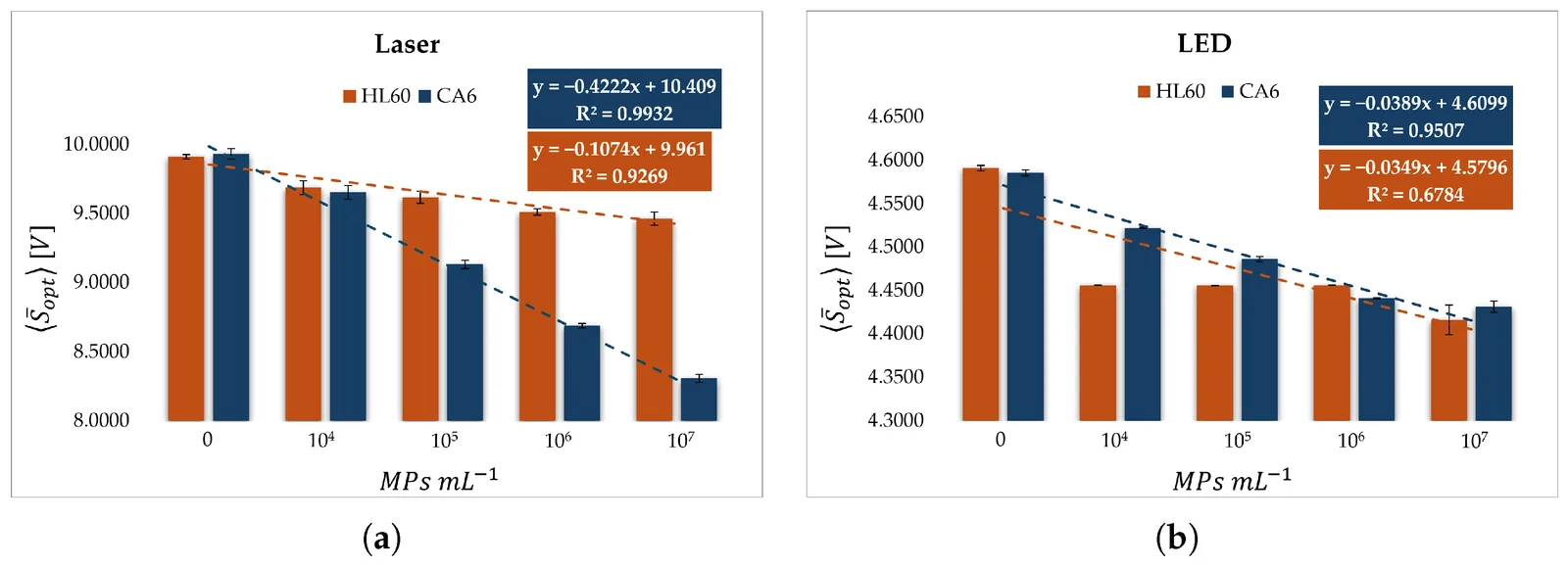
Concentration-dependent optical response of HL60 cells and CA6 microparticles under (**a**) laser and (**b**) LED illumination. The bars show the mean optical signal over three repeated acquisitions ($\langle {\bar{S}}_{\mathrm{opt}}\rangle$), while the error bars represent the corresponding standard error of the mean (SEM). Dashed lines indicate the linear regressions across the investigated concentration levels.

Figure 8 shows the mean optical response obtained for HL60 cells and CA6 microparticles at the five investigated concentration levels, including the particle-free reference condition. The bars represent the mean of the three repeated acquisitions ($\langle {\bar{S}}_{\mathrm{opt}}\rangle$), whereas the error bars indicate the corresponding standard error of the mean (SEM). For both employed illumination sources, the detected voltage generally decreased as the microparticle concentration increased, consistent with the working principle illustrated in Figure 2.

Under laser illumination (see Figure 8a), both microparticle types exhibited a clear concentration-dependent response. For HL60 cells, the mean signal decreased from 9.9120V in the particle-free condition down to 9.4628V at $10^{7}\;\mathrm{cells}\;{\mathrm{mL}}^{-1}$, corresponding to an overall reduction of approximately 4.5%. A considerably greater variation was observed for CA6 microparticles, for which the signal decreased from 9.9326 down to 8.3049V, corresponding to a reduction of approximately 16.4%. The empirical linear regressions calculated over the concentration levels yielded slopes of −0.1074 for HL60 cells and −0.4222 for CA6 microparticles, with coefficients of determination of $R^{2}=0.9269$ and $R^{2}=0.9932$, respectively. The magnitude of the CA6 slope was therefore approximately four times that obtained for HL60 cells, quantitatively confirming the greater sensitivity of the detected optical signal to variations in CA6 concentration. These results indicate that the laser configuration provided a consistent concentration-dependent response for both microparticle types, with a substantially more pronounced variation for the synthetic microparticles.

The different signal reductions observed for the two microparticle classes confirm the particle-dependent optical behavior predicted by the ray-tracing simulations presented in Section 3.1. To provide a direct comparison, the experimental signals at the simulated concentration of $3\times 10^{5}\;\mathrm{MPs}\;{\mathrm{mL}}^{-1}$ were estimated by interpolation on the logarithmic concentration scale between $10^{5}$ and $10^{6}\;\mathrm{MPs}\;{\mathrm{mL}}^{-1}$. The resulting values were approximately 9.568V for HL60 cells and 8.919V for CA6 microparticles, corresponding to a 6.8% signal reduction from HL60 to CA6. This result is consistent with the approximately 8.0% reduction in the total collected optical flux predicted by the ray-tracing model for CA6 relative to HL60. The close agreement between the simulated and experimental variations supports the interpretation that the detected response is governed by the combined physical and optical properties of the microparticle suspension.

A lower signal amplitude and a reduced concentration-dependent variation were observed under LED illumination (see Figure 8b). The HL60 signal decreased from 4.5907V in the particle-free condition down to 4.4161V at $10^{7}\;\mathrm{cells}\;{\mathrm{mL}}^{-1}$, corresponding to an overall reduction of approximately 3.8%. After the initial decrease at $10^{4}\;\mathrm{cells}\;{\mathrm{mL}}^{-1}$, the response remained nearly constant between $10^{4}$ and $10^{6}\;\mathrm{cells}\;{\mathrm{mL}}^{-1}$. Accordingly, the empirical linear regression yielded a slope of −0.0349 and a relatively low coefficient of determination of $R^{2}=0.6784$. For CA6 microparticles, the signal progressively decreased from 4.5853 down to 4.4312V, with an overall reduction of approximately 3.4%. In this case, the regression yielded a slope of −0.0389 and a higher coefficient of determination of $R^{2}=0.9507$. Thus, for CA6 microparticles, the LED configuration preserved a clearer inverse relationship between concentration and transmitted signal, although with lower sensitivity than the laser configuration.

The different responses obtained with the two illumination sources may be attributed to their distinct emission characteristics and coupling efficiencies. The monochromatic and directional laser beam produced a higher detected signal and more pronounced concentration-dependent variations. Conversely, LED illumination produced a lower detected signal and less pronounced variations. This effect was particularly evident for HL60 cells, whose translucent nature and relatively low optical contrast with the suspending medium produced only weak variations in the transmitted LED signal. Therefore, under the adopted experimental configuration, the spatially coherent and directional laser illumination provided the highest sensitivity to concentration variations and the most effective discrimination between microparticle types.

### 3.3. Analysis of Variance (ANOVA): Effects of Experimental Factors

A three-way analysis of variance (ANOVA) was performed to quantify the effects of microparticle type, concentration, and illumination source on the detected optical signal.

The investigated response (dependent variable) of the experimental design is the mean optical signal, denoted as $\langle {\bar{S}}_{\mathrm{opt}}\rangle$.

The investigated factors (independent variables) of the experimental design are:

- *Microparticle type (factor A)*—Categorical factor varied between two levels (a=2), corresponding to HL60 cells and CA6 microparticles;
- *Concentration (factor B)*—Categorical factor varied among five levels (b=5), corresponding to 0, $10^{4}$, $10^{5}$, $10^{6}$, and $10^{7}\;\mathrm{MPs}\;{\mathrm{mL}}^{-1}$;
- *Light source (factor C)*—Categorical factor varied between two levels (c=2), corresponding to laser and LED illumination.

For each combination of the three factors, three repeated acquisitions were performed (R=3), resulting in a balanced full-factorial experimental design comprising a×b×c×R=2×5×2×3=60 observations. Although concentration was defined by numerical values, it was treated as a categorical fixed factor in the ANOVA because its levels were logarithmically spaced and included the particle-free condition. Statistical significance was evaluated at α=0.05. The investigated experimental plan is reported in Table 4.

The ANOVA results are summarized in Table 5. All three main factors significantly affected the detected optical signal. In particular, a significant effect was found for microparticle type (*F*-value = 412.46, *p*-value < 0.001), concentration (*F*-value = 329.13, *p*-value < 0.001), and illumination source (*F*-value = 173,312.63, *p*-value < 0.001).

The significant microparticle-type effect confirms that HL60 cells and CA6 microparticles produced different optical responses, consistent with both the experimental trends and the ray-tracing results. Similarly, the significant concentration effect confirms that the detected signal varied systematically with the number of microparticles within the optical interrogation volume. The particularly large light-source effect, as confirmed by *F*-value = 173,312.63, primarily reflects the different signal levels produced by the laser and LED configurations. Since the two sources generated different particle-free baseline voltages, this main effect should not be interpreted independently as a direct measure of concentration sensitivity.

All two-way interactions were also statistically significant. The type–concentration interaction (*F*-value = 96.97, *p*-value < 0.001) indicates that the concentration-dependent variation differed between HL60 cells and CA6 microparticles. This result is consistent with the markedly different slopes measured under laser illumination. The type–light source interaction (*F*-value = 477.33, *p*-value < 0.001) shows that the difference between the two microparticle types depended on the selected illumination source. Indeed, laser illumination produced a clear separation between HL60 and CA6, whereas their LED slopes were considerably smaller and more similar.

The significant concentration–light source interaction (*F*-value = 188.41, *p*-value < 0.001) further demonstrates that the effect of concentration depended on the illumination configuration. This agrees with the more pronounced concentration-dependent signal variations observed with the laser compared with the LED. Finally, the significant three-way interaction (Type × Concentration × Light source, *F*-value = 88.66, *p*-value < 0.001) indicates that the different concentration responses of HL60 and CA6 were themselves dependent on the illumination source. Therefore, the effects of microparticle type, concentration, and illumination source cannot be interpreted independently.

In conclusion, the ANOVA results quantitatively confirm the trends identified in the concentration-response curves. Microparticle concentration significantly affected the detected signal, but the magnitude of this effect depended on both microparticle type and illumination source. In particular, the laser configuration provided the greatest sensitivity to concentration variations and the clearest particle-dependent response, while the LED produced weaker and more similar responses for the two microparticle classes.

## 4. Conclusions

This work proposed a micro-optofluidic platform for the label-free and in-situ estimation of microparticle concentration within microchannels. By integrating optical-fiber interfaces for light delivery and collection directly into the device, the proposed approach eliminates the need for microscope-based sample observation during sensing. It is therefore particularly suitable for embedded and multilayer lab-on-chip platforms in which the microfluidic layer is enclosed within the device and direct optical access is limited.

The platform was investigated through complementary numerical, experimental, and statistical analyses. Ray-tracing simulations were used to examine light propagation and particle-induced scattering within the optical interrogation volume. Experimental validation was performed using biological HL60 cells and synthetic CA6 microparticles at five concentration levels, ranging from the particle-free condition to $10^{7}\;\mathrm{MPs}\;{\mathrm{mL}}^{-1}$, under laser and LED illumination. Finally, a three-way ANOVA assessed the influence of microparticle type, concentration, and illumination source on the detected optical signal.

The simulations predicted a particle-dependent optical response, with an approximately 8.0% lower collected flux for CA6 microparticles than for HL60 cells at the same concentration. This result was consistent with the experimental response, which showed an estimated difference of 6.8% between the two microparticle types at the corresponding concentration. Experimentally, the detected signal generally decreased with increasing concentration for both suspensions. Laser illumination exhibited the highest concentration sensitivity, with an overall signal reduction of 4.5% for HL60 cells and 16.4% for CA6 microparticles over the investigated concentration range. Conversely, LED illumination preserved a concentration-dependent trend for CA6 microparticles, although with lower sensitivity than the laser, whereas only weak and non-uniform variations were observed for HL60 cells.

The statistical analysis confirmed that microparticle type, concentration, and illumination source significantly affected the detected signal. Their two- and three-way interactions were also significant (*p*-value < 0.001), demonstrating that the concentration-dependent response was determined both by microparticle properties and illumination conditions.

Overall, the agreement among numerical, experimental, and statistical results establishes the proposed platform as a reliable proof of concept for label-free, in-situ detection of concentration-dependent variations in both biological and synthetic microparticle suspensions.

Future developments will focus on coherent illumination, which demonstrated the highest concentration sensitivity in the present study. In particular, the wavelength dependence of the optical response will be investigated to determine how particle scattering and transmitted-signal attenuation vary with the optical properties of biological and synthetic microparticles. Additionally, the approach will be extended to microparticles and cells with different size, shape, rigidity, and optical properties. Finally, the approach will be evaluated in multilayer devices containing embedded microchannels that are not externally accessible to conventional imaging. Such configurations represent a particularly relevant application of the proposed in-situ sensing approach, as optical interrogation can be performed directly within the device without requiring visual access to the microfluidic layer.

## Figures and Tables

**Table 1:** Comparison between representative integrated optofluidic approaches for microparticle detection and the platform proposed in this work.

Optical Configuration	Sample and Flow Regime	Detection Mode	Measured Output	Main Limitations for Concentration Monitoring	References
Integrated excitation and collection fibers	Particles and biological objects in continuous flow	Label-free	Individual scattering events	Requires single particle focusing and controlled passage through the interrogation region	Mohan et al. [50]; Storti et al. [51]
Multichannel optical-absorption detection	Particles distributed among parallel microchannels	Label-free	Individual absorption events	Requires particle separation and multiple parallel detection channels	Xian et al. [53]
Multiple on-chip excitation and collection fibers	Cells or beads encapsulated within droplets	Label-free scattering; label-based fluorescence	Single-particle scattering and fluorescence	Requires droplet generation and multiple collection paths; fluorescence analysis requires labeling	Gupta et al. [52]
All-fiber separation and counting system	Microparticles in continuous flow	Label-based	Particle separation and counting	Requires elasto-inertial separation and fluorescent particles	Kumar et al. [54]
Fiber-coupled dynamic light scattering or extinction spectroscopy	Nano- and microparticle suspensions	Label-free	Particle-size distribution and concentration	Requires multiple collection angles or optical path lengths, correlation analysis, or spectroscopic instrumentation	Chen et al. [55]; Onofri et al. [56]
Ti-indiffused waveguides integrated into lithium niobate	Plastic microspheres within flowing water droplets	Label-free	Transmitted-light intensity	Requires droplet generation and a specialized lithium-niobate platform; evaluated on synthetic particles	Bragato et al. [57,58]
Opposing fibers coupled to a PDMS microchannel	HL-60 cells and CA6 microparticles in continuous single-phase flow	Label-free	Mean transmitted signal for concentration estimation	Bulk measurement without single-particle counting; particle-specific calibration is required	This work

**Table 2:** Physical and optical properties of the dispersed phases (microparticles or cells) and corresponding carrier fluids used in this study.

	Dispersed-Phase Properties				Carrier-Fluid Properties		
Dispersed Phase	Diameter (μm)	*ρ_p_* (g cm^−3^)	*n_p_*	Roughness (nm)	Carrier Fluid	*ρ_m_* (g cm^−3^)	*n_m_*
**CA6** (PMMA microparticles)	∼6	1.20	1.492	30–60	Glycerol–DI water (80:20, v/v)	∼1.20	∼1.450
**HL60** cells	∼15	1.05	1.361	∼5	Phosphate-buffered saline (PBS)	∼1.00	∼1.334

*Note.* $\rho_{p}$ and $n_{p}$ denote the density and refractive index of the dispersed phase, respectively; $\rho_{m}$ and $n_{m}$ denote the corresponding properties of the carrier fluid.

**Table 3:** Summary of the experimental campaign. R = 3 replicated acquisitions of 60s were performed for each experimental condition.

Parameter	HL60 Cells	CA6 Microparticles
Concentration ($MPs\;{\mathrm{mL}}^{-1}$)	$0,\;10^{4},\;10^{5},\;10^{6},\;10^{7}$	
Illumination source	Laser; LED	
Flow rate	$0.008\;\mathrm{mL}\;\min^{-1}$	

**Table 4:** Three-way Analysis of Variance (ANOVA) experimental plan: investigated factors and corresponding levels. Three repeated acquisitions were performed for each combination of factor levels.

Factor	Symbol	Type	Unit	Levels	Level I	Level II	Level III	Level IV	Level V
Microparticle type	A	Categorical	[–]	a=2	HL60	CA6	[–]	[–]	[–]
Concentration	B	Categorical	$\mathrm{MPs}\;{\mathrm{mL}}^{-1}$	b=5	0	$10^{4}$	$10^{5}$	$10^{6}$	$10^{7}$
Light source	C	Categorical	[–]	c=2	Laser	LED	[–]	[–]	[–]

**Table 5:** Three-way ANOVA results for the detected optical signal as a function of microparticle type, concentration, and illumination source.

Source	*SS*	*df*	*MS*	*F*-Value	*p*-Value
Microparticle type	0.8593	1	0.8593	412.46	<0.001
Concentration	2.7427	4	0.6857	329.13	<0.001
Light source	361.0605	1	361.0605	173,312.63	<0.001
Type × Concentration	0.8081	4	0.2020	96.97	<0.001
Type × Light source	0.9944	1	0.9944	477.33	<0.001
Concentration × Light source	1.5700	4	0.3925	188.41	<0.001
Type × Concentration × Light source	0.7388	4	0.1847	88.66	<0.001
Error	0.0833	40	0.0021	–	–
Total	368.8572	59	–	–	–

*Note.* *SS*: Sums of Squares; *df*: degrees of freedom; *MS*: Mean Squares.

## Data Availability

Dataset available on request from the authors.
